# Microbial dysbiosis in systemic lupus erythematosus: a scientometric study

**DOI:** 10.3389/fmicb.2024.1319654

**Published:** 2024-05-28

**Authors:** Miaomiao Zhao, Xiaoting Wen, Ruiling Liu, Ke Xu

**Affiliations:** ^1^Third Hospital of Shanxi Medical University, Shanxi Bethune Hospital, Shanxi Academy of Medical Sciences, Tongji Shanxi Hospital, Taiyuan, China; ^2^Shanxi Bethune Hospital, Shanxi Academy of Medical Sciences, Tongji Shanxi Hospital, Third Hospital of Shanxi Medical University, Taiyuan, China; ^3^Department of Microbiology and Immunology, Basic Medical College, Shanxi Medical University, Jinzhong, China

**Keywords:** systemic lupus erythematosus, autoimmune disease, microbiota, bibliometric analysis, VOSviewer

## Abstract

**Introduction:**

Systemic lupus erythematosus (SLE) is a chronic autoimmune disease. Mounting evidence suggests microbiota dysbiosis augment autoimmune response. This study aims to provide a systematic overview of this research field in SLE through a bibliometric analysis.

**Methods:**

We conducted a comprehensive search and retrieval of literature related to microbial researches in SLE from the Web of Science Core Collection (WOSCC) database. The retrieved articles were subjected to bibliometric analysis using VOSviewer and Bibliometricx to explore annual publication output, collaborative patterns, research hotspots, current research status, and emerging trends.

**Results:**

In this study, we conducted a comprehensive analysis of 218 research articles and 118 review articles. The quantity of publications rises annually, notably surging in 2015 and 2018. The United States and China emerged as the leading contributors in microbial research of SLE. Mashhad University of Medical Sciences had the highest publication outputs among the institutions. Frontiers in Immunology published the most papers. Luo XM and Margolles A were the most prolific and highly cited contributors among individual authors. Microbial research in SLE primarily focused on changes in microbial composition, particularly gut microbiota, as well as the mechanisms and practical applications in SLE. Recent trends emphasize “metabolites,” “metabolomics,” “fatty acids,” “T cells,” “*lactobacillus*,” and “dietary supplementation,” indicating a growing emphasis on microbial metabolism and interventions in SLE.

**Conclusion:**

This study provides a thorough analysis of the research landscape concerning microbiota in SLE. The microbial research in SLE mainly focused on three aspects: microbial dysbiosis, mechanism studies and translational studies (microbiota-based therapeutics). It identifies current research trends and focal points, offering valuable guidance for scholars in the field.

## 1 Introduction

Systemic lupus erythematosus (SLE) is a chronic, systemic autoimmune disorder characterized by abnormal activation of T and B lymphocytes, production of autoantibodies, complement system activation, and the formation of immune complexes (Zucchi et al., 2022). Its primary clinical characteristics encompass the involvement of multiple systems and organs, along with recurrent relapses and remissions (Kiriakidou and Ching, 2020). Individuals afflicted with SLE present a wide array of symptoms and disease progression. Predominant manifestations include fever, fatigue, malar rash, photosensitivity, myalgia or arthralgia, arthritis, and renal complications (Goldblatt and O’Neill, 2013). Furthermore, SLE patients exhibit an elevated susceptibility to atherosclerosis, thrombosis, arteritis, embolism, and vascular spasm. The gravest consequences of SLE are infections and severe multisystemic damage, particularly affecting the nervous system and kidneys (Gordon et al., 2018). SLE is strikingly dominated by women of childbearing age, with the ratio of women to men being 10:1. The prevalence and severity of SLE exhibit considerable variation across different regions and ethnic backgrounds (Fanouriakis et al., 2021).

The precise etiology of SLE remains incompletely elucidated. The pathogenesis of SLE may be attributed to genetic, hormonal, and environmental factors, including infections, medications, and exposure to UVA light (Barbhaiya and Costenbader, 2016). With the rapid advancement of next-generation sequencing technology, our understanding of microorganisms has deepened. The human body harbors approximately 10^14 microorganism species. The human microbiota includes bacteria, fungi, viruses, archaea, and protozoa, bacteria being the predominant microorganisms, which is mainly composed of four phyla (*Proteobacteria*, *Actinobacteria*, *Firmicutes*, and *Bacteroidetes*) (Tong et al., 2020). Studies of microbiota dysregulation have highlighted dysbiosis as a significant internal environmental factor associated with SLE. Apperloo-Renkema et al. (1994) initially demonstrated that modifications in the composition of intestinal microbiota could induce SLE in animal models. This phenomenon is likely attributed to a compromised defense mechanism of indigenous gut microbes against exogenous bacteria (Apperloo-Renkema et al., 1994). Persistent antigenic stimulation could instigate alterations in the intestinal microecology, resulting in immune system dysregulation. Consequently, the immune system may erroneously target self-tissues through the production of antibodies or sensitized lymphocytes. Amplified inflammatory responses further exacerbate the production of these antibodies, culminating in a spectrum of clinical manifestations and subsequent complications associated with SLE (Zhang and Reichlin, 2008).

In recent years, the role of microbiota in the pathogenesis of SLE has garnered widespread attention, resulting in an exponential increase in related publications. While there have been review and research articles on microorganisms and SLE, a lack of trend analysis in this research area is evident. Bibliometrics is a multidisciplinary science that utilizes mathematical and statistical methods to quantitatively analyze all forms of knowledge, thereby reflecting the knowledge structure and developmental characteristics of a scientific field. Bibliometric analysis is advantageous in identifying and describing subtle differences and evolutions within a scientific domain. It has already been extensively applied in fields such as economics (Peng et al., 2023), management (de Sousa, 2021), information science (Wang et al., 2023), energy and the environment (Ola et al., 2023). Consequently, this paper employs bibliometric methods to systematically analyze the research landscape of microorganisms and SLE in the medical field, to provide historical context and predict the current hot topics and emerging areas within this field.

## 2 Materials and methods

### 2.1 Data sources and search strategy

**Figure 1:**
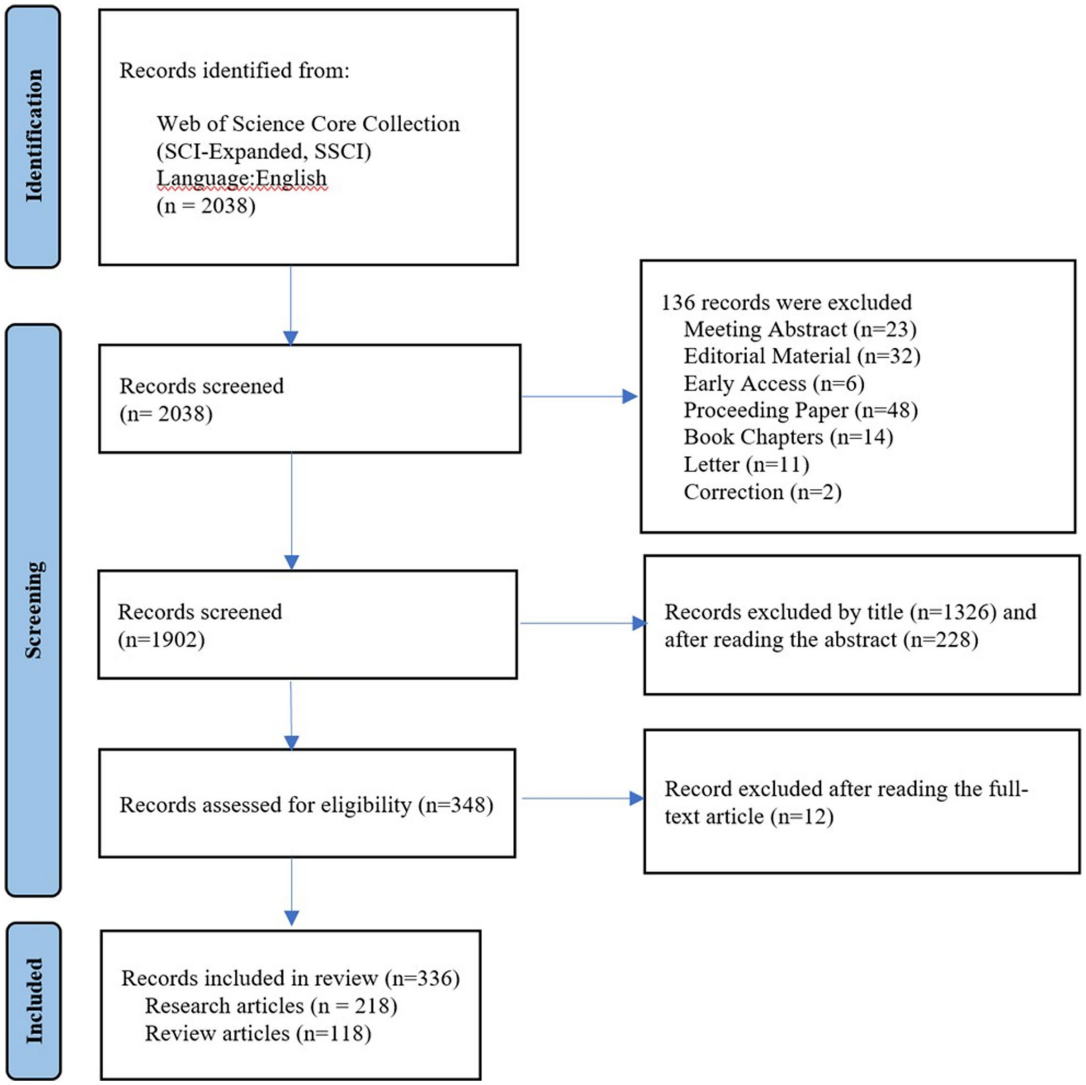
The study PRISMA flow diagram.

In this study, we utilized the Web of Science Core Collection (WoSCC) database to retrieve relevant literature on microorganisms and SLE published between 1991 and 2022. The WoSCC is a collection of high-quality academic resources available on the Web of Science™ platform, commonly used for literature retrieval, journal selection, research evaluation, and bibliometric analysis. To ensure minimal bias resulting from database updates, literature search and retrieval were conducted on the same day. The search strategy included synonymous terms for microorganisms and SLE, with Boolean logic set as (TS = (microbiome OR microbiota OR microbe OR microorganism OR flora OR microflora OR germ OR bacteri* OR Microbial metabolism OR Dietary Supplements OR probiotics OR Prebiotics OR Synbiotics OR Fecal Microbiota Transplantation) AND TS = (systemic lupus erythematosus) AND LA = (English)). The above-mentioned keywords were selected from the Medical Subject Headings (MeSH) provided by the National Library of Medicine (NLM)/PubMed. The data were exported as plain text files, which included full records and cited references. The article types were set as “Research Article” and “Review Article” to assess the trends and hot topics in research on microorganisms and SLE. Initially, a total of 2038 articles were retrieved through the search. However, after manually excluding irrelevant content related to the research topic, 336 relevant articles remained. The exclusion of irrelevant articles was conducted based on careful examination and consideration of the research focus. Only articles that were directly related to the study of microorganisms and SLE were included in the final set of results. Articles selection process was presented in a PRISMA flowchart (Figure 1).

### 2.2 Bibliometrics analysis and data visualization

VOSviewer is a software tool that creates visualizations of network data, enabling the exploration and analysis of these visualizations (van and Waltman, 2010). In this study, VOSviewer (v1.6.18.0) was utilized to generate co-occurrence networks, temporal maps, and density maps of keywords based on the research literature on microorganisms and SLE from the WoSCC database. Additionally, national and institutional collaboration networks were visualized. Microsoft Excel 2021 was employed to analyze publication trends, while the bibliometrix (Aria and Cuccurullo, 2017) was used to analyze other information such as authorship, journals, and countries’ publication outputs. These methods were employed to identify the hot topics and research trends in this field, providing valuable insights and references for future studies.

## 3 Results

### 3.1 Quantities and trend of publications output

**Figure 2:**
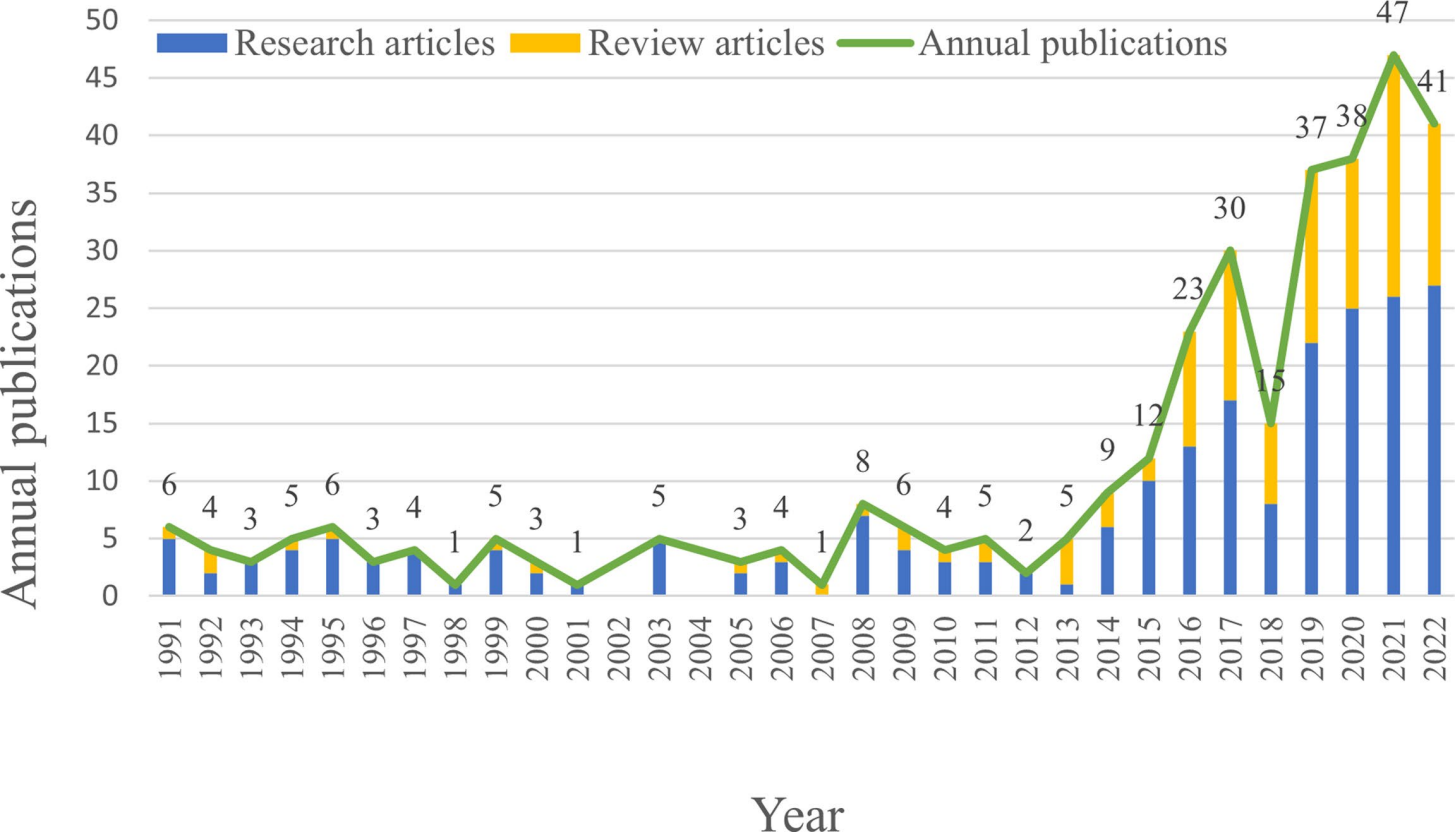
Quantities of publications output of the microbial research in SLE from 1991 to 2022.

Publication output is an important indicator for assessing the research hotspot and the progress made in the field. In this study, a total of 336 articles related to the microbial research in SLE were included, consisting of 218 Research articles and 118 Review articles (Figure 2). The annual publication output of microbial research in SLE is depicted in Figure 2. It showed that the earliest Research articles and the first Review article were published in 1991. From 1991 to 2014, the annual production was relatively stable (less than 10 articles), with minor fluctuations. From 2015 to 2021, the publication output significantly increased to 47 articles, though there was a sharp decline in 2018. It suggested that the microbial research in SLE has gained increasing attention.

### 3.2 Analysis of countries/regions and affiliations

**Figure 3:**
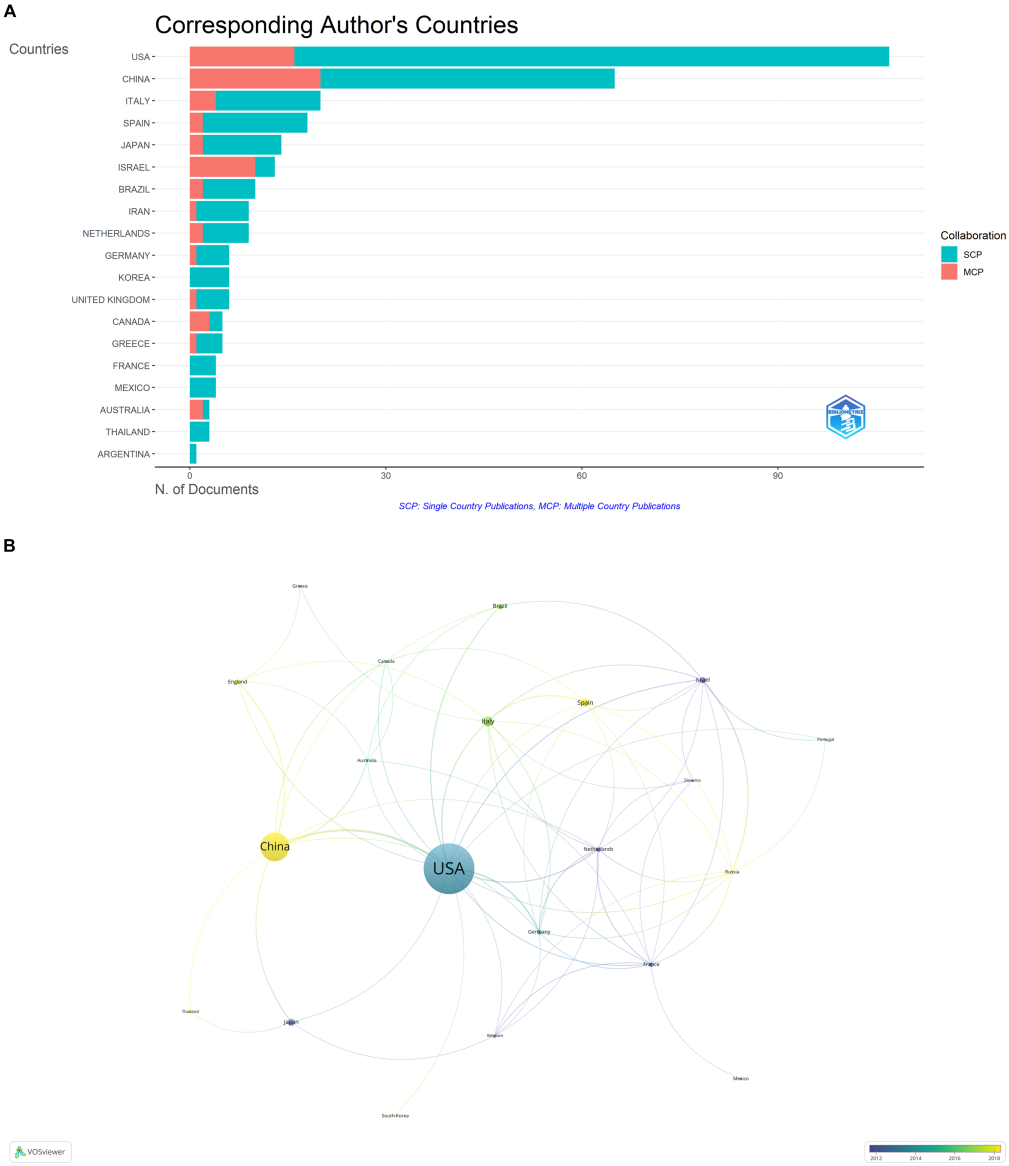
Analysis of publications on microbial research in SLE by the countries/regions. **(A)** Visualization of papers according to the country of the corresponding authors. MCP, Multiple Country Publications; SCP, Single Country Publications. **(B)** Visualization of papers according to the cooperation networks of the corresponding authors.

**Figure 4:**
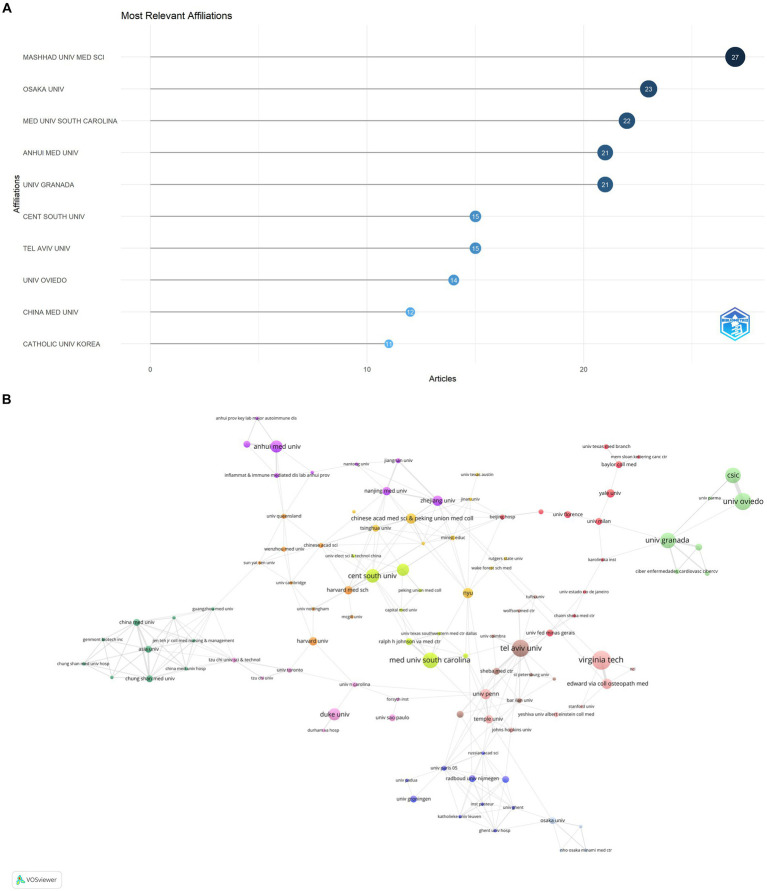
Analysis of publications on microbial research in SLE by the affiliations. **(A)** Visualization of papers according to the affiliations. **(B)** Visualization of papers according to the cooperation networks of the affiliations.

The publications on microbial research in SLE are contributed by 34 countries/regions. Table 1 displayed the top 10 countries in the number of publications. The United States ranked first, accounting for 37.80% (127/336) of the total publications, followed by China with 21.13% (71/336). Italy and Spain also made substantial contributions. The proportion of Multiple Country Publications (MCP) reflects the level of international collaboration and academic exchange. It showed that most countries/regions, conducted academic researches by themselves, but there was also international collaboration to a certain degree. The publication numbers did not correlate to international collaboration in terms of author collaborations (Figure 3A). Notably, more than half of Israeli publications resulted from international collaborations, though its publication number ranked sixth. The cooperation network of the countries/regions illustrated that international collaboration was mainly carried out by countries/regions actively involved in this field (Figure 3B). The collaboration between China and the United States was the most prominent. When calculating the average citations per article (total citations/total number of publications) for each country, the top three countries were Germany (57.30 citations), Spain (51.60 citations), and the United States (46.00 citations) with more than 40 citations, while the average citations of France and Japan were less 10. Among the top 10 institutions (Figure 4), Mashhad University of Medical Sciences, Osaka University, Medical University of South Carolina, Anhui Medical University, and University of Granada had made notable contributions for more than 20 articles of each institution (Figure 4A). The collaborations among affiliations were primarily based on countries (Figure 4B). Tel Aviv University engaged in extensive and productive collaboration with various institutions, despite having fewer publications compared to Mashhad University of Medical Sciences, whose publication outputs were the most. Three Chinese institutions, Anhui Medical University, Central South University, and China Medical University, listed in the top 10 institutions in terms of publication outputs. Moreover, Central South University (15 publications) had extensive collaboration with the Medical University of South Carolina (22 publications) from the United States.

**Table 1:** The top 10 countries/regions in the microbial research of SLE.

Country	Number of publications	Total citations	Average article citations
USA	127	4,927	46.00
China	71	1,379	21.20
Italy	26	487	24.40
Spain	21	929	51.60
Japan	17	129	9.20
Israel	16	470	36.20
Brazil	15	354	35.40
Germany	13	344	57.30
Netherlands	12	355	39.40
France	12	38	9.50
England	10	175	29.20

Average article citations = total citations/total number of publications.

### 3.3 Analysis of authors and journals

**Figure 5:**
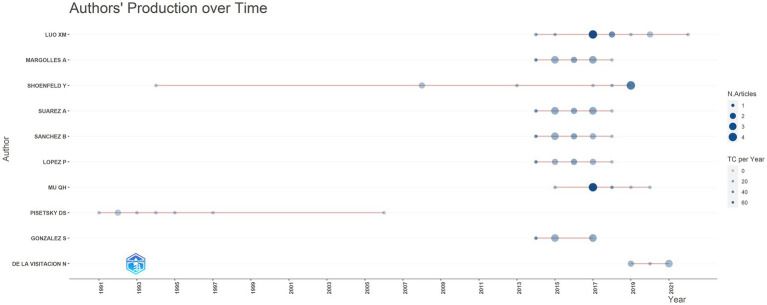
Analysis of publications on microbial research in SLE by the authors.

Since 1991, a total of 1,675 scholars dedicated to research on this topic. Table 2 and Figure 5 presented the top 10 authors with the highest publication number and their citation counts. Luo XM, Margolles A and Shoenfeld Y were the top three authors in terms of publication outputs and had the greatest academic impact, with total citation counts of 990, 685, and 766, respectively. Pisetsky DS achieved remarkable achievements early in this field, publishing a total of 7 papers between 1991 and 1997. Shoenfeld Y published one article during this period. In the past decade, an increasing number of authors have focused on the potential role of microbiota in SLE. Among them, Luo XM had the most publication outputs and citation counts. His team primarily investigates the dysbiosis of gut microbiota and potential mechanisms in SLE, as well as the therapeutic effects of bacteria.

**Table 2:** The top 10 authors in the microbial research of SLE.

Element	Articles	h-index	Total citations	Year of Publication_start
Luo XM (Zhang et al., 2014; Mu et al., 2015, 2017a,b, 2019, 2020; Edwards et al., 2017; Abdelhamid and Luo, 2018; Luo et al., 2018; Abdelhamid et al., 2020; Cabana-Puig et al., 2022; Christovich and Luo, 2022)	12	10	990	2014
Margolles A (Hevia et al., 2014a, 2015; Cuervo et al., 2015; Rojo et al., 2015; Sánchez et al., 2015; López et al., 2016a,b; González et al., 2017; Rodríguez-Carrio et al., 2017a; Ruiz et al., 2018)	10	8	685	2014
Shoenfeld Y (Baharav et al., 1994; Amital et al., 2008; Doria et al., 2008; Pordeus et al., 2008; Bogdanos et al., 2013; Chen et al., 2017; Ceccarelli et al., 2018; De Luca and Shoenfeld, 2019; Nogueira and Shoenfeld, 2019; Shoenfeld et al., 2019)	10	8	766	1994
Suarez A (Hevia et al., 2014a,b; Cuervo et al., 2015; Rojo et al., 2015; López et al., 2016a,b; González et al., 2017; Rodríguez-Carrio et al., 2017a; Ruiz et al., 2018)	9	8	762	2014
Sanchez B (Cuervo et al., 2015; Hevia et al., 2015; Rojo et al., 2015; Sánchez et al., 2015; López et al., 2016a,b; González et al., 2017; Rodríguez-Carrio et al., 2017a; Ruiz et al., 2018)	9	8	711	2014
Lopez P (Hevia et al., 2014a,b; Cuervo et al., 2015; Rojo et al., 2015; López et al., 2016a,b; González et al., 2017; Rodríguez-Carrio et al., 2017a; Ruiz et al., 2018)	9	8	468	2014
Mu QH (Mu et al., 2015, 2017a,b, 2019, 2020; Edwards et al., 2017; Gutiérrez-Díaz et al., 2018; Luo et al., 2018)	8	8	790	2015
Pisetsky DS (Gilkeson et al., 1991; Robertson et al., 1992; Robertson and Pisetsky, 1992; Bunyard and Pisetsky, 1994; Gilkeson et al., 1995; Wu et al., 1997; Hamilton et al., 2006; Mu et al., 2017c)	8	7	599	1991
Gonzalez S (Pisetsky, 1997; Hevia et al., 2014a; Cuervo et al., 2015; Rojo et al., 2015; Sánchez et al., 2015; González et al., 2017; Rodríguez-Carrio et al., 2017a,b; de la Visitación et al., 2019, 2020, 2021a,b)	7	6	197	2014
De La VISITACION N (Rodríguez-Carrio et al., 2017b; de la Visitación et al., 2019, 2020, 2021a,b,c)	6	6	662	2019

WOS h-index: The articles of authors and journals published are sorted in descending order based on the number of citations. Each of these articles has been cited at least H times, while the citation counts of other articles do not exceed H. This delineates the h-index of the authors and the journals.

Academic journals serve as crucial platforms for showcasing scientific research accomplishments. We conducted an analysis using 336 papers published in 171 different journals. Table 3 presented the top 10 journals in terms of publication counts, which published a total of 114 papers, accounting for 33.93% (114/336) of the total publications. Most of these journals focused on the field of immunology. Frontiers in immunology and Lupus had the highest publication counts, 42 and 19 papers, respectively. Frontiers in Immunology (Impact Factor = 8.786) was the most cited journal. Although Annals of Rheumatic Diseases ranked eighth, it had the highest Impact factor (2021) of 28.003.

**Table 3:** The top 10 journals in the microbial research of SLE.

	Journal	Articles	h-index	Impact factor-2021	Total citations	Start year
1	Frontiers in Immunology	42	20	8.786	1,162	2015
2	Lupus	19	11	2.858	384	1994
3	Journal of Autoimmunity	10	7	14.551	389	2008
4	Autoimmunity Reviews	8	6	17.39	195	2006
5	International Journal of Molecular Sciences	7	6	6.208	384	1991
6	Clinical and Experimental Immunology	6	6	5.732	180	2014
7	Current Opinion in Rheumatology	6	12	5.006	358	2008
8	Frontiers in Microbiology	6	12	6.064	381	2016
9	Annals of Rheumatic Diseases	5	12	28.003	108	2019
10	Clinical Immunology	5	12	10.19	118	2019

WOS h-index: The articles of authors and journals published are sorted in descending order based on the number of citations. Each of these articles has been cited at least H times, while the citation counts of other articles do not exceed H. This delineates the h-index of the authors and the journals.

### 3.4 High-cited articles

We conducted a citation analysis on the 336 articles. In bibliometrics, Global Citations (GCS) refers to the total number of citations that an article has received in the WoSCC database, while Local Citations (LCS) refers to the number of citations within our dataset, reflecting the impact in the field of microbial research in SLE. Table 4 depicted the top 10 highly cited articles within our dataset. The original research articles “10.1128/mBio.01548-14” and “10.1128/AEM.02676-14” received high global citations and local citations, indicating their significant influence in the field.

**Table 4:** The top 10 high-cited articles in the microbial research of SLE.

No.	Document	DOI	Title	Component	Year	LCS	GCS
1	HEVIA A, 2014, MBIO	10.1128/mBio.01548-14	Intestinal Dysbiosis Associated with Systemic Lupus Erythematosus	Research article	2014	120	350
2	ZHANG HS, 2014, APPL ENVIRON MICROB	10.1128/AEM.02676-14	Dynamics of Gut Microbiota in Autoimmune Lupus	Research article	2014	81	173
3	LUO XM, 2018, APPL ENVIRON MICROB	10.1128/AEM.02288-17	Gut Microbiota in Human Systemic Lupus Erythematosus and a Mouse Model of Lupus	Research article	2018	65	149
4	MU QH, 2017, MICROBIOME	10.1186/s40168-017-0300-8	Control of lupus nephritis by changes of gut microbiota	Research article	2017	61	160
5	HE ZX, 2016, GUT PATHOG	10.1186/s13099-016-0146-9	Alterations of the gut microbiome in Chinese patients with systemic lupus erythematosus	Research article	2016	59	136
6	AZZOUZ D, 2019, ANN RHEUM DIS	10.1136/annrheumdis-2018-214856	Lupus nephritis is linked to disease-activity associated expansions and immunity to a gut commensal	Research article	2019	53	164
7	ZEGARRA-RUIZ DF, 2019, CELL HOST MICROBE	10.1016/j.chom.2018.11.009	A Diet-Sensitive Commensal *Lactobacillus* Strain Mediates TLR7-Dependent Systemic Autoimmunity	Research article	2019	45	141
8	LOPEZ P, 2016, SCI REP-UK	10.1038/srep24072	Th17 responses and natural IgM antibodies are related to gut microbiota composition in systemic lupus erythematosus patients	Research article	2016	44	129
9	JOHNSON BM, 2015, CLIN EXP IMMUNOL	10.1111/cei.12609	Impact of dietary deviation on disease progression and gut microbiome composition in lupus-prone SNF1 mice	Research article	2015	43	92
10	MU QH, 2017, SCI REP-UK	10.1038/s41598-017-14223-0	Antibiotics ameliorate lupus-like symptoms in mice	Research article	2017	35	67

GCS represents the total citations of an article in WOSCC, while LCS represents the number of citations in our collections.

### 3.5 Keywords co-occurrence network

**Figure 6:**
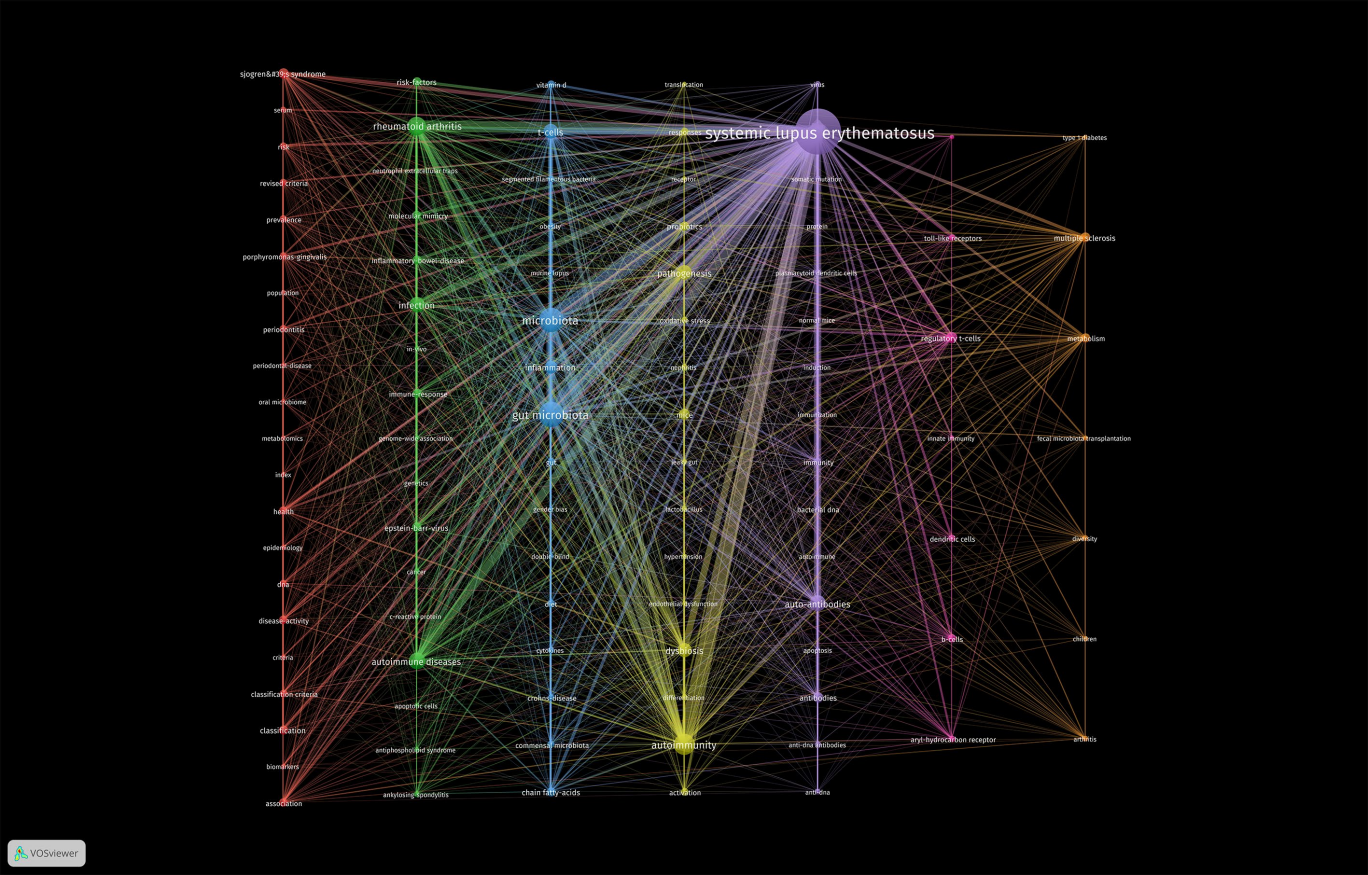
Cluster visualization of the co-occurrence network according to the authors’ keywords. The minimum number of occurrences of the author’s keywords was set to 5. Finally, 101 keywords were included in the co-occurrence network. These keywords were divided into seven different clusters.

Keywords are highly concise summaries of the content of a literature. Thus, keyword co-occurrence network analysis performed using VOSviewer software can indicate the research scope and hotspots in the field, aiding to the discovery of research trends. To ensure readability and visual appeal of the graphs, a minimum occurrence threshold of 5 was set for the keywords in these papers. Then 101 keywords were produced and divided into six clusters represented by different colors (Figure 6). These six clusters exhibited overlapping and intersecting characteristics, indicating that researches in this field were not dispersed or isolated.

As shown in Figure 6, the purple cluster was the largest, which meant the research core in this field, encompassing keywords such as “bacterial DNA”, “virus”, “autoantibodies”, “dendritic cells”, and “autoimmunity”. The second largest cluster, blue cluster contained 15 nodes, including “gut microbiota”, “lupus mice”, “inflammation”, “diet”, “fatty acids”, “segmented filamentous bacteria” and “T cells”. These two clusters primarily explored the relationship between microbiota and SLE.

The green cluster reflected researchers’ exploration of the pathogenetic effect of microbial dysbiosis in other autoimmune diseases like “rheumatoid arthritis,” “inflammatory bowel disease,” “antiphospholipid syndrome” and “ankylosing spondylitis.”

The yellow, orange, and pink clusters displayed researches on gut microbiota in SLE disease progression. They mainly focused on two aspects: firstly, the regulation of gut microbiota, including terms “dysbiosis,” “*lactobacillus*,” “dietary supplements,” “fecal microbiota transplantation” and “metabolites”; secondly, immunological pathogenesis involving terms such as “leaky gut,” “toll-like receptors,” “regulatory T cells,” “dendritic cells,” “B cells” and “innate immunity.”

The red cluster mainly encompassed keywords related to oral microbial alterations and relevant experimental researches in SLE. The key terms were “oral microbiota,” “periodontitis” and “periodontal disease”. Additionally, “classification system,” “disease activity” and “metabolomics” were also frequent keywords in this cluster, indicating that microbiota may induce oral disease of SLE patients through metabolites.

### 3.6 Hotspots and topic migration

**Figure 7:**
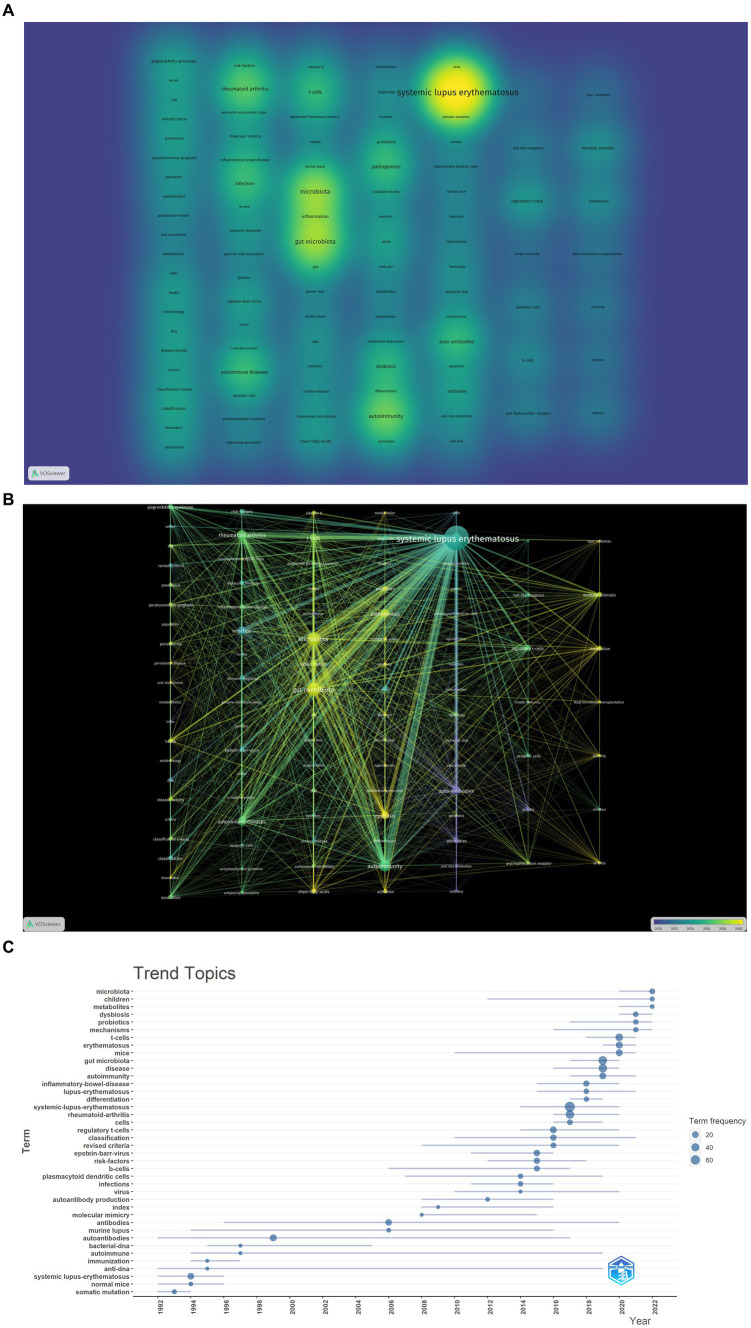
Analysis of hotspot and topic migration on microbial research in SLE. **(A)** The density visualization of keywords co-occurrence network (the figure represented the numbers of keyword occurrences). **(B)** The timestamp visualization of keywords co-occurrence network. The co-occurrence network was based on time characteristics, and each node was color-coded with a different color based on the average time multiple of the keywords. **(C)** The historical migration of research hotspots.

The density visualization of the co-occurrence network of key terms is depicted in Figure 7A. The research content of these articles revolves around “SLE,” “autoimmunity” and “gut microbiota,” highlighting the pivotal role of immune and inflammatory responses in SLE. T cell-mediated cellular immunity stands out as a prominent area of research. Mechanistically, particular attention is directed toward intestinal permeability, the production of short-chain fatty acids by gut microbiota, and the impact of gut microbiota on T cells.

In the timeline graph of keyword co-occurrence (Figure 7B), each node is color-coded based on the average temporal multiplier of the corresponding keyword. It can be observed that high-frequency keywords exhibit an average onset time post-2015, indicating the emergence of a novel field. As demonstrated in Figure 7B, the recent research emphasis predominantly centers on the mechanistic exploration of gut microbiota involvement in SLE and the investigation of applications such as fecal microbiota transplantation.

We conducted a “trend topic” analysis employing bibliometric software packages for comprehending the historical dynamics of research hotspots in this domain. As depicted in Figure 7C, during the initial phase, the scholarly output in this field was significantly limited. Preceding 2013, there existed inadequate continuity among high-frequency keywords. In 2014, 2015, and 2016, prominent keywords encompassed “B cells,” “dendritic cells,” “infection,” “viruses” and “regulatory T cells.” This suggests that the pathogenetic research of microorganisms primarily focused on the functions of immune cells in SLE. In 2017 and 2018, high-frequency keywords included “rheumatoid arthritis” and “inflammatory bowel disease” which are also autoimmune diseases related to immune function and microorganisms. These diseases share common mechanisms and pathways, providing new insights for the microbial study in SLE. Since 2019, frequent keywords such as “gut microbiota,” “mechanisms,” “T cells,” “dysbiosis” and “metabolites” have appeared, indicating that the recent research mainly focused on the mechanisms and applications of gut microbiota in SLE, which is consistent with the results shown in Figure 7B.

## 4 Discussion

### 4.1 Overview of development in the field of microbiota in SLE

In this study, we retrieved 218 research articles and 118 review articles about the microbial study in SLE from 1991 to 2022 in the WOSCC database. The annual publications were steady from 1991 to 2014. From 2015 significant attention was paid to this field. In addition to the development of microbial sequencing, such as 16S rRNA gene sequencing and metagenomic sequencing, this may be attributed to increasing interest in bacteria-induced immune response in autoimmune disease. The greatest contributors were the United States and China. Two institutions from the two countries, the Medical University of South Carolina and Central South University had intensive collaboration. Luo XM, Margolles A, and Shoenfeld Y had the greatest impact in this field. Frontiers In Immunology had the most publication numbers. Notably, the impact factors of the top 10 journals were higher than 5, indicating the high quality of articles. “10.1128/mBio.01548-14” and “10.1128/AEM.02676-14” had the highest LCS. The highly cited articles used sequencing technology to reveal microbial dysbiosis mainly in the gut as well as the pathogenesis of SLE.

### 4.2 Structure, current status, and direction of microbial research in SLE

In about 20 years, the research on microbiota in SLE mainly focused on three aspects. The first was searching for evidence of microbial dysbiosis to SLE. The second was mechanism studies, involving “molecular simulation,” “Toll-like receptors,” “T cells” and “fatty acids.” The third was translational studies using probiotic supplements, dietary interventions and targeted microbiota-based therapeutics.

#### 4.2.1 Microbiota dysbiosis in SLE

The activity and composition of the microbiota are influenced by genetic background, age, diet, and the overall health status of the host. In turn, the composition and activity of the microbiota also affect the host metabolism and the development of diseases (Manos, 2022). The composition and the dynamic patterns of microbiome dysbiosis can serve as early indicators of SLE onset (Rahbar Saadat et al., 2019). The researches mainly focused on gut microbiota. Additionally, the dysbiosis in oral, skin and plasma also received increasing interest.

##### 4.2.1.1 Gut microbiota

In “10.1128/mBio.01548-14,” 16S sequencing and metagenomic sequencing were performed on fecal samples from SLE patients and healthy individuals, revealing a significant decrease in the Firmicutes/Bacteroidetes ratio and overexpression of polysaccharide degradation pathways in the microbiota of SLE patients. “10.1128/AEM.02676-14” analyzed differences in gut microbiota in a lupus-prone mouse model and found a significant decrease in *Lactobacillaceae* and significant enrichment of *Clostridiaceae* family XIII and *Streptococcaceae* in lupus-prone mice, with gender differences observed in the gut microbiota changes.

The gut microbiota plays a role in the development and regulation of the immune system, potentially influencing autoimmunity by modulating the balance between tolerance and inflammatory microorganisms (Dominguez-Bello et al., 2010; Lee and Mazmanian, 2010). Currently, various mouse models have been applied to understand gut microbiota dysbiosis in SLE. Apperloo-Renkema et al. (1995) were the first to find that the antimicrobial colonization quality of the gut microbiota reduced in active SLE patients, which may lead to increased translocation of foreign bacteria, then facilitate the production of anti-double-stranded DNA (anti-dsDNA) autoantibodies. Compared to healthy individuals, non-active SLE patients have a lower abundance of the *phylum Firmicutes*, as well as a lower *Firmicutes/Bacteroidetes* (F/B) ratio (Hevia et al., 2014a). Additionally, SLE patients show a decrease in *Ruminococcaceae* and *Lachnospiraceae*, and a significant increase in *Prevotellaceae, Bacteroidaceae*, and intestinal *Streptococcus* (Hevia et al., 2014a). Furthermore, *Streptococcus pneumoniae* and *Streptococcus intermedius* (both belonging to the normal microbiota in the oral cavity and gastrointestinal tract) are increased in SLE patients, indicating a potential association between the microbiota-mediated oral-gut axis and the pathological changes in SLE (Tomofuji et al., 2021). Zhang et al. (2014) discovered significant reductions in *Lactobacilli* and increased abundance of *Lachnospiraceae* in the gut microbiota of MRL/lpr mice. Furthermore, the abundance of *Lachnospiraceae* was closely associated with the disease progression, while the colonization of *Lactobacilli* in the gut showed a negative correlation with lupus activity. These findings suggest that the gut microbiota is involved to some extent in the development of SLE.

##### 4.2.1.2 Oral microbiota

Keywords cluster showed oral microbiota was a hotspot in SLE. When the disease affects the oral mucosa, patients often experience symptoms such as dry mouth, insufficient saliva production, oral ulcers, and periodontal disease. The oral microbiome forms a highly interconnected microbial community network and can distribute throughout the human body (Avila et al., 2009). Guo et al. (2023) discovered that an increased abundance of *Prevotella* and *Alloprevotella* of the *Prevotellaceae* family, *Leptotrichia* of the *Fusobacteriaceae* family, *Veillonella* and *Megasphaera* of the *Veillonellaceae* family in the oral microbiome of SLE patients. Additionally, the abundance of 15 bacteria, including members of the *Micrococcaceae, Bacillaceae,* and *Abiotrophia,* were decreased. Furthermore, with increasing disease activity in SLE, the abundance of *Abiotrophia* and *Lactobacillus* increases, while the abundance of *Phyllobacterium* and Micrococcaceae decreases. Even the dysregulation of subgingival microbiota in SLE patients was linked to periodontal conditions and can result in a 1.76-fold higher risk of developing periodontitis (Rutter-Locher et al., 2017). SLE patients with concurrent periodontitis exhibit increased abundance of *Prevotella nigrescens*, *Prevotella oulorum, Prevotella oris, Selenomonas noxia, Lachnospiraceae,* and *Leptotrichia* in both inflamed and healthy sites (Corrêa et al., 2017). The presence of pathogenic bacteria is positively correlated with the systemic inflammatory levels in SLE patients, and patients with concurrent periodontitis show elevated IL-6, IL-17, and IL-33 (Marques et al., 2016). It is worth noting that SLE patients exhibit an enrichment of certain microorganisms in the intestinal tract, including *A. massiliensis, S. satelles,* and *A. rimae*, which are closely associated with oral inflammation. This suggests that the microbiota enriched in the intestinal tract of SLE patients may originate from the oral cavity (Chen et al., 2021). Thus alterations in the oral microbiota may lead to the dysbiosis of gut bacteria and cause inflammation in SLE.

##### 4.2.1.3 Skin microbiota

Around 80% of SLE patients experienced cutaneous manifestations and up to 25% of patients presented with skin involvement as the initial symptom (Yell et al., 1996). SLE patients exhibit dysbiosis of the skin microbiota, characterized by an increased proportion of *Staphylococcus* and *Corynebacterium* in the lesional skin and a decreased abundance of *Cutibacterium acnes* (Zhou et al., 2021). Compared to healthy skin, SLE patients show significant reductions in the abundance of *Prevotella, Rothia* and *Klebsiella* in the lesional skin. Furthermore, there is a significant decrease in the abundance of *Acidobacteria*, *Gemellaceae* and *Corynebacterium* in both the affected and unaffected skin of SLE patients (Huang et al., 2020). In addition, the microbial community diversity of SLE lesions is associated with clinical features of the patients, including gender, renal involvement, low serum complement levels, and myositis. Disruption of the skin microbiome may have the potential for SLE diagnosis, with a combination of *Staphylococcus aureus*, *Staphylococcus epidermidis* and *Staphylococcus hominis* showing high accuracy and serving as microbial biomarkers for SLE diagnosis (Huang et al., 2020).

##### 4.2.1.4 Plasma microbiota

The dysfunction of the skin, mucosa or gut barrier will increase permeability and allow microbe and/or their products to translocate into the system, causing excessive and persistent immune stimulation. Lipopolysaccharide (LPS) is a representative marker of microbial translocation (Marchetti et al., 2013). SLE patients and their first-degree relatives exhibit elevated plasma levels of LPS, which is positively correlated with anti-dsDNA antibodies, indicating a higher degree of microbial translocation in both patients and relatives (Ogunrinde et al., 2019). Compared to healthy controls, both SLE patients and their first-degree relatives show a decrease in the abundance of *Paenibacillus* in the blood. Furthermore, the enrichment of *Desulfoconvexum, Desulfofrigus, Desulfovibrio, Draconibacterium, Planococcus*, and *Psychrilyobacter* in SLE patients is directly associated with increased plasma autoantibodies levels (Schett et al., 2002; Reveille, 2004). *Planococcus* is increased in the plasma of SLE patients and can stimulate peripheral blood mononuclear cells (PBMCs) to produce pro-inflammatory cytokines (Luo et al., 2020).

#### 4.2.2 Potential mechanisms of microbiota in SLE

The relationship between microbial dysbiosis and SLE remains unclear. Although genetic susceptibility is an important factor contributing to dysbiosis of the microbiota in lupus-prone mice and the progression of autoimmune diseases, environmental factors appear to be more crucial than host genetics (Ulff-Møller et al., 2018). Microbial dysbiosis can lead to the development of SLE through various mechanisms.

##### 4.2.2.1 Microorganisms interact with the host immune system

The microbiota plays a vital role in shaping and maturing the host’s immune system, while the immune system reciprocally maintains crucial aspects of host-microbe symbiosis. The maturation and stability of microbiota, especially the gut microbiota, occur in parallel with the development of the immune system (Arpaia et al., 2013). Even after development ceases, the microbiota continues to interact with the host immune system. The host maintains a host-microbe symbiotic equilibrium by limiting tissue inflammation and microbial translocation by reducing microbial contact with epithelial cell surfaces. The intestinal mucosal epithelial barrier serves as a defense against the invasion of foreign toxins. Disruption of the gut microbiota community homeostasis can result in alterations in paracellular transport, cell apoptosis, or increased intercellular permeability, ultimately leading to “intestinal permeability” or “leaky gut” (Christovich and Luo, 2022). Additionally, certain bacteria and their metabolic byproducts can induce T cell differentiation and proliferation of T helper (Th17) cells. The RORγt+ T regulatory cells (Treg) cell subset is stimulated by short-chain fatty acids (SCFAs) derived from microbiota, specifically *Clostridia* (Arpaia et al., 2013). The colonization of *segmented filamentous bacteria* (SFB) can promote TH17 cell expansion (Ivanov et al., 2009). RORγt+ Treg cells and Th17 cells are crucial for controlling immune responses to gut microbiota, maintaining the gut barrier, reducing inflammation and lessening certain immune reactions mediated by Th2 cells (Yang et al., 2016; Pandiyan et al., 2019). In SLE patients, fecal samples demonstrate a significant increase in calprotectin (a biomarker of impaired intestinal barrier function), while serum levels of soluble CD14, α1-acid glycoprotein, and LPS are elevated, indicating compromised intestinal barrier function and gut bacterial translocation (Azzouz et al., 2019). Microbial dysbiosis can result in an imbalance of Treg/Th17 responses in patients with SLE, instigating immune reactions and facilitating the production of autoantibodies (Rother and van der, 2015; Pandiyan et al., 2019). Moreover, diminished levels of Treg cells can give rise to the generation of bacterial antigens, eliciting Th17-Th1 effector responses against beneficial bacteria, and promoting the development of inflammatory symbiotic reactions and autoimmune responses (Ivanov et al., 2008).

##### 4.2.2.2 Epitope spreading and molecular mimicry

Autoantibodies are expanded induced by bacteria through epitope spreading and molecular mimicry. Anti-Ro60 antibodies are the most common and earliest preclinical antinuclear antibodies in SLE. Commensal Ro60 homologs can trigger autoimmune responses through cross-reactivity in the human body (Greiling et al., 2018). Immunization with Ro60 protein can also lead to the production of anti-Ro52, anti-La, anti-Sm, and anti-U1-RNP antibodies through intermolecular epitope spreading in mice (Deshmukh et al., 1999). Additionally, *Escherichia coli* can induce the production of RNA and dsDNA antibodies and promote the immune response against β2-glycoprotein I (Manfredo Vieira et al., 2018; Salem et al., 2019). *Fretibacterium, Lachnospiraceae, Prevotella,* and *Selenomonas* can also activate the autoimmune response through cross-reactivity with self-antibodies and contribute to the development of SLE (Corrêa et al., 2017; Mu et al., 2017a). Zhang and Reichlin (2008) discovered that antigens from *Burkholderia* can bind to dsDNA antibodies in the serum of SLE patients. Some peptides from *Odoribacter splanchnicus* and *Akkermansia muciniphila* share high similarity with Sm antigen and Fas antigen epitopes (Chen et al., 2021). The amino acid residues 35–58 of Epstein–Barr virus nuclear antigen-1 (EBVNA-1) have similar cross-reactive epitopes with Sm and Ro, leading to SLE-like diseases (Lossius et al., 2012).

##### 4.2.2.3 Microbial biofilms and metabolites

Bacterial biofilms primarily composed of starch-binding amyloid proteins, accumulation of amyloid protein/DNA complexes can trigger intracellular DNA sensors, TLR9, stimulating immune cascade reactions that lead to the transcription of type I IFN and the production of anti-nuclear antibodies (di Domizio et al., 2012). Curli fibers in *S. typhimurium* can also bind to bacterial DNA, activate dendritic cells to secrete pathogenic type I IFN, stimulate the proliferation of activated T cells, B cells, and inflammatory monocytes (Gallo et al., 2015).

Microbial metabolites are involved in a wide range of physiological processes in the human body, can act as signaling molecules and substrates for metabolic reactions, modulating host immune function (Nicholson et al., 2012; Wang et al., 2019). Substances such as SCFAs- butyrate (Kaisar et al., 2017; Gonçalves et al., 2018), tryptophan (Mohammadi et al., 2018; Choi et al., 2020), polyamines (Sánchez-Jiménez et al., 2019) and lactic acid (Mu et al., 2017a; Lee et al., 2018) can enhance or regulate host immune tolerance through diverse pathways, such as reducing pro-inflammatory cytokines, maintaining immune tolerance, preserving mucosal barrier integrity, and modulating intestinal immune cells. The reduction of *Firmicutes* and augmentation of *Bacteroidetes* in SLE patients result in decreased SCFAs production, thereby exacerbating inflammatory responses (Katz-Agranov and Zandman-Goddard, 2021). Resistant starch can promote the digestion of fiber by the gut microbiota into SCFAs, which has been shown to reduce the abundance of *Lactobacillus reuteri*, alleviate lupus-like symptoms, downregulate the type I interferon (IFN) pathway, and decrease lupus-related mortality (Zegarra-Ruiz et al., 2019). Elevated trimethylamine N-oxide (TMAO), derived from gut microbiota choline metabolism, may serve as a risk factor for concurrent atherosclerosis in SLE mice (González-Correa et al., 2021). Therefore, it is evident that the homeostasis of human microbiota and its metabolic products are crucial for normal physiological activities of the body.

#### 4.2.3 Application potential of microbiota in SLE

Research on gut microbiota intervention therapy for SLE is still in its early stages, yet lessons can be gleaned from other dysbiosis-related disorders, enabling the prediction of future therapeutic approaches for SLE.

##### 4.2.3.1 Fecal microbiota transplantation

Fecal microbiota transplantation (FMT) involves the transplantation of gut microbiota from a healthy donor to a patient’s gastrointestinal tract, either through the upper or lower digestive pathways, to restore microbial balance and improve various gastrointestinal dysfunctions. FMT has been proven to be an effective, safe, and cost-efficient method for treating recurrent Clostridioides difficile infection, with a clinical success rate exceeding 90% (Lee et al., 2016). Experimental evidence suggests that transplantation of gut microbiota from SLE-prone mice to germ-free C57BL/6 mice results in changes in the distribution of immune cells in the recipients and upregulation of lupus-susceptible genes (Ma et al., 2019). The decreased abundance of gut microbial genera, such as Ruminococcus and Alistipes, in the gut microbiota of prednisone-treated MRL/lpr mice indicates that FMT may mediate the effects of hormones such as prednisone (Wang et al., 2021). In SLE patients, FMT therapy leads to a reduction in pro-inflammatory microbial groups (*Porphyromonas, Pseudomonas genera and Alphaproteobacteria class, Prevotella and Veillonella genera,* and *the Burkholderiales order*) and an increase in SCFA-producing bacterial taxa (*phylum Firmicutes, including Eubacterium hallii group, Dorea, Marvinbryantia,* and *Papillibacter*) (Huang et al., 2022). This suggests that FMT contributes to the alleviation and improvement of systemic inflammation and clinical symptoms in SLE patients.

##### 4.2.3.2 Probiotics, prebiotics, and synbiotics

Probiotics, prebiotics, and synbiotics can maintain microbial balance by influencing immune homeostasis, promoting the production of various nutrients, degrading toxic compounds, and generating antimicrobial compounds (Li et al., 2020). In lupus patients, probiotics and prebiotics can induce the differentiation of Treg cells, improve the imbalance of Th17/Th1, and reduce the production of autoantibodies, thereby alleviating the severity of the disease (Zhang et al., 2021). A mixture composed of five strains of lactobacilli (*Lactobacillus oris, Lactobacillus rhamnosus, Lactobacillus reuteri, Lactobacillus johnsonii,* and *Lactobacillus gasseri*) can repair intestinal permeability, decrease the production of intestinal IL-6, and increase the generation of IL-10, promoting the formation of an anti-inflammatory environment (Mu et al., 2017a). Additionally, the probiotic *Lactobacillus fermentum* CECT5716 (LC40) (Toral et al., 2019) and/or the *bifidobacterium Bifidobacterium breve* CECT7263 can prevent TLR9-induced hypertension and endothelial dysfunction in a mouse model of erythematous pustulosis (de la Visitación et al., 2021a). *Bifidobacteria* can prevent excessive activation of CD4+ T cells, thus maintaining the balance of Treg, Th17, and Th1 cells in SLE patients (López et al., 2016a). SLE patients exhibit increased serum levels of high-sensitivity C-reactive protein (CRP), which is associated with an increased risk of cardiovascular disease (Barnes et al., 2005; Rezaieyazdi et al., 2011). Widhani et al. (2022) found that synbiotics effectively reduce the elevated levels of high-sensitivity CRP in SLE patients, increase the *Firmicutes/Bacteroidetes* (F/B) ratio and butyrate metabolism, and decrease nucleotide sugar and amino sugar metabolism, thereby reducing disease activity. *Lactic acid bacteria*, as probiotics, can modulate immune and anti-inflammatory responses by reducing interleukin-6 (IL-6) and enhancing IL-10 levels. Supplementation of *Lactic acid bacteria* can also decrease proteinuria and levels of autoantibodies, improve renal pathology scores in MRL/lpr mice, reduce inflammatory cytokines, increase anti-inflammatory cytokine levels, and enhance the population of Tregs (Mu et al., 2017a).

##### 4.2.3.3 Dietary intervention

The influence of diet on autoimmune diseases should not be underestimated. Consumption of a high-salt diet has the potential to activate dendritic cells (DCs) and stimulate the production of pathogenic Th17 cells via the p38/MAPK-STAT1 signaling pathway. This activation can result in dysbiosis of the gut microbiota, further exacerbating immune system dysregulation and worsening SLE (Kleinewietfeld et al., 2013; Zhang et al., 2021). Dietary modifications can impact the composition of the gut microbiota. Incorporating a moderate intake of protein, along with supplementation of vitamins, minerals, polyunsaturated fatty acids (PUFAs), and plant estrogens, can aid in improving patients’ immune function, regulating systemic inflammation, and reducing disease activity. These dietary interventions have the potential to slow down the progression of SLE (Vieira et al., 2014; Putri et al., 2022).

##### 4.2.3.4 Symbiotic microbial consortia

Symbiotic microbial consortia aim to assemble bacterial strains with favorable characteristics into beneficial microbiome components for the host organism. Tvede and Rask-Madsen cultivated a consortium comprising 10 strains of facultative and obligate anaerobic bacteria. They administered colonic therapy to five individuals with recurrent infections of the challenging pathogen *Clostridioides difficile* (*C. difficile*). None of the five patients experienced recurrence, indicating a certain efficacy of the consortium (Tvede and Rask-Madsen, 1990). A six-strain symbiotic bacterial consortium successfully eradicated *C. difficile* from the intestines of infected mice (Lawley et al., 2012). Further experiments revealed that a four-strain consortium, featuring *Clostridium scindens*, which possesses the unique capacity to convert primary bile acids into secondary bile acids known to inhibit *C. difficile* growth, significantly bolstered resistance to *C. difficile* colitis in mice (Buffie et al., 2015). Research on symbiotic microbial consortia in SLE is limited and primarily focused on animal models. While these consortia (*Lactobacillus oris, Lactobacillus rhamnosus, Lactobacillus reuteri, Lactobacillus johnsonii, and Lactobacillus gasseri*) can control the progression of lupus nephritis, the primary actors in this process are *L. reuteri* and an uncultured *Lactobacillus* sp. (Mu et al., 2017a). The application of microbial consortia lies in uncovering correlations between different microbial community compositions or microbial-encoded metabolic pathways and experimental or clinical phenotypes. Given the vast diversity of commensal microbial species in the human body and the multitude of potential combinations of symbiotic species, such research poses significant challenges. The development of machine learning and artificial intelligence platforms undoubtedly will propel advancements in this field.

##### 4.2.3.5 Engineered symbiotic bacteria

Currently, the capacity to engineer bacterial strains residing in the gastrointestinal tract also holds potential practical value. Advancements in genetic engineering techniques have facilitated significant upregulation of recombinant protein expression in *Bacteroidales* strains, achieving up to a 9,000-fold increase using the 16S ribosomal RNA gene promoter and the TetR repressor (Lim et al., 2017). Expansion of genetically manipulable *Bacteroidales* strains has been achieved through gain-of-function vector construction, enabling allelic replacement and utilization of specific polysaccharides (García-Bayona and Comstock, 2019). *Bacteroides thetaiotaomicron* has been successfully utilized for heterologous expression of tryptophan decarboxylase from *R. gnavus*, leading to enhanced tryptamine production in the lower intestinal tract (Bhattarai et al., 2018). Subsequent investigations have shown that colonization of mice with this recombinant *B. thetaiotaomicron* strain augments mucus release from goblet cells and enhances resistance to dextran sulfate sodium-induced colitis (Bhattarai et al., 2020). Research on engineered symbiotic microorganisms remains at the laboratory stage due to factors such as technological limitations and safety considerations.

The pathogenesis and organ manifestations of SLE are intricately linked to dysbiosis of the microbiota, underscoring the prospective utilization of microbiota-based interventions in the management of SLE. Contemporary investigations propose that alterations in the gut microbiota have the potential to modulate the development of SLE; however, the precise underlying mechanisms remain elusive. Consequently, further research endeavors are imperative to elucidate the intricate interplay between the microbiota and SLE. Moreover, treatment strategies should adopt a comprehensive approach, considering both genetic and environmental factors.

## 5 Conclusion

Over the previous decade, a remarkable upsurge in scholarly publications has occurred, attracting researchers and institutions from diverse countries/regions. Notably, China and the United States have emerged as the most active contributors in this domain. Recent research endeavors have predominantly focused on exploring the phenomenon of microbiota dysbiosis, specifically pertaining to gut microbiota dysbiosis, alongside elucidating the intricate mechanisms and applications associated with SLE, thereby establishing it as a prominent area of investigation. Future research in this field can be oriented toward several pivotal directions. Firstly, additional studies are warranted to ascertain the relationship between the microbiota and SLE, as well as to unravel the precise underlying mechanisms. Secondly, prospective research initiatives are essential to monitor the dynamic fluctuations of the microbiota and identify distinct patterns of microbiota alterations that correlate with the development of SLE. Lastly, through investigating the intricate interactions between the microbiota and SLE, potential therapeutic targets can be discerned, paving the way for the development of personalized treatment strategies targeting the microbiome.

## Data availability statement

The original contributions presented in the study are included in the article/supplementary material, further inquiries can be directed to the corresponding author.

## Author contributions

MZ: Data curation, Formal analysis, Methodology, Software, Visualization, Writing – original draft. XW: Data curation, Formal analysis, Funding acquisition, Methodology, Supervision, Writing – review & editing. RL: Data curation, Formal analysis, Methodology, Writing – review & editing. KX: Conceptualization, Data curation, Funding acquisition, Investigation, Methodology, Project administration, Resources, Supervision, Writing – review & editing.
